# SFMBT2 hypermethylation promotes colorectal cancer progression and is a potential noninvasive biomarker for advanced CRC

**DOI:** 10.1016/j.isci.2026.115019

**Published:** 2026-02-13

**Authors:** Wei Wang, Ling Yu, Wenyuan He, Jingyi Wu, Yuansen Li, Wenzhi Cui, Danli Ye, Juan Zhou, Ming Xie, Xuwen Lai, Chengyong Lei

**Affiliations:** 1 Department of Pathology, General Hospital of Southern Theater Command, People’s Liberation Army of China, Guangzhou 510010, China; 2 Department of Urology, Nanfang Hospital, Southern Medical University, Guangzhou 510515, China; 3 Guangzhou University of Chinese Medicine, Guangzhou, Guangdong, China; 4 Department of Oncology, General Hospital of Southern Theater Command, People’s Liberation Army of China, Guangzhou 510010, China; 5 Department of Pathology, The First Naval Hospital of Southern Theater Command, People’s Liberation Army of China, Zhanjiang 510010, China

**Keywords:** Diagnostics, Cancer

## Abstract

The SFMBT2 protein is a Polycomb group protein implicated in transcriptional regulation and cancer biology. Here, we investigated the role of *SFMBT2* promoter hypermethylation in colorectal cancer (CRC) development and its clinical relevance. Using CRC cell lines, FFPE tissues, plasma samples, and public datasets, we show that promoter hypermethylation is associated with the transcriptional silencing of *SFMBT2*. *SFMBT2* expression was high in normal intestinal mucosa but progressively reduced in advanced adenoma, primary CRC, and metastatic lesions, accompanied by increased promoter methylation. Elevated plasma *SFMBT2* methylation was associated with higher recurrence risk in stage III CRC and poor prognosis in stage IV disease. Bioinformatic analyses further linked high *SFMBT2* expression to enhanced immune cell infiltration and activation of immune-related pathways. These findings identify *SFMBT2* hypermethylation as a potential noninvasive biomarker for recurrence risk stratification and prognostic assessment in advanced CRC, and suggest a role for *SFMBT2* in shaping the tumor immune microenvironment.

## Highlights

• *SFMBT2* promoter hypermethylation drives transcriptional silencing in CRC
• *SFMBT2* expression decreases from adenoma to metastatic CRC
• Plasma *SFMBT2* methylation predicts recurrence and prognosis in advanced CRC
• *SFMBT2* expression correlates with immune infiltration and immune pathways

## Graphical abstract

**Figure:**
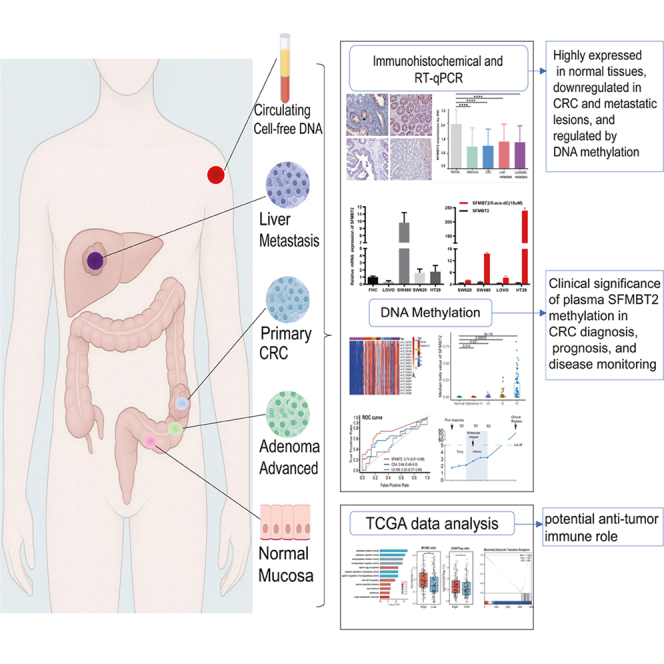

## Introduction

Aberrant DNA hypermethylation is a well-established epigenetic mechanism driving tumorigenesis and progression through the transcriptional silencing of tumor suppressor genes in various cancers.^1^^,^^2^^,^^3^^,^^4^ Extensive research has identified hypermethylated promoters of key suppressor genes such as *MGMT, SFRP2, SEPT,* and *P16* in CRC.^5^^,^^6^^,^^7^^,^^8^ In our prior biomarker discovery research for early detection and recurrence risk assessment in CRC, *SFMBT2* emerged as a promising candidate.^9^^,^^10^ *SFMBT2*, an epigenetically regulated gene encoding a transcriptional repressor that binds methylated histone H3 and H4 tails, is frequently silenced by hypermethylation in various cancers.^11^^,^^12^ This silencing promotes cancer progression, such as facilitating epithelial-mesenchymal transition (EMT) and metastasis in prostate cancer through the dysregulation of key pathways (Wnt, TGF-β, and Notch).^13^ Additionally, *SFMBT2* hypermethylation-induced silencing has been implicated in the pathogenesis of breast, lung, gastric, renal, and esophageal cancers by inactivating tumor-suppressive pathways.^14^^,^^15^^,^^16^^,^^17^ Moreover, the involvement of *SFMBT2* hypermethylation in early CRC detection has been validated, thereby reinforcing its potential as a diagnostic and prognostic biomarker.^18^^,^^19^ This study aims to elucidate the role of *SFMBT2* promoter hypermethylation in CRC pathogenesis and its clinical utility by quantifying methylation levels in tissue and plasma samples across CRC stages, and evaluating the value for stratifying recurrence risk and prognostic assessment. In addition, basic functional studies were conducted to characterize the tumor-suppressive mechanisms.

## Results

### Biological significance of *SFMBT2* expression in colorectal cancer tissues and human colorectal cancer cells

**Figure 1:**
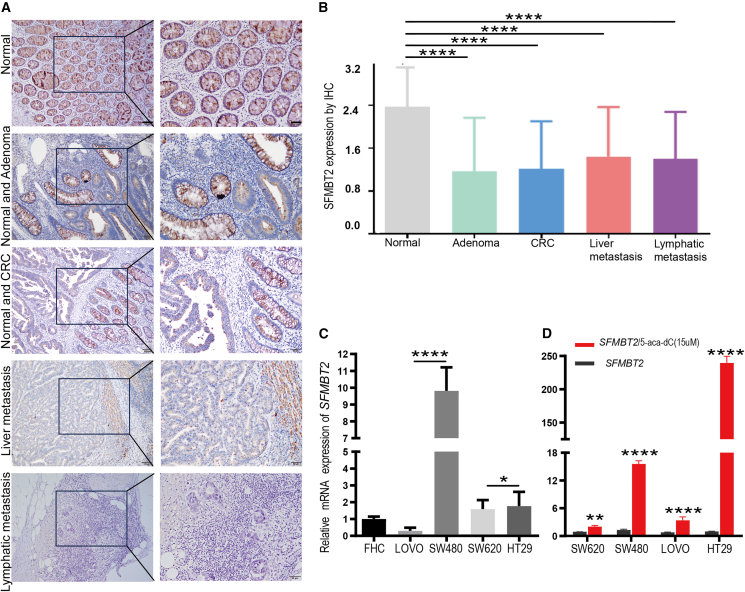
SFMBT2 expression patterns in colorectal cancer cell lines and tissues (A) Representative immunohistochemical staining shows high SFMBT2 protein expression in normal intestinal mucosa, with markedly reduced or absent expression in AA, primary CRC tissues, and distant metastatic lesions. Scale bars, 100 μm, 50 μm (B) Quantitative analysis revealed a statistically significant decrease in SFMBT2 expression across disease progression stages (*p* < 0.001). (C) Baseline expression of SFMBT2 mRNA was high in FHC (normal intestinal epithelial cells), HT29, and SW480 cell lines, but low in Lovo and SW620 cells. (D) SFMBT2 mRNA expression was assessed in four CRC cell lines (SW620, SW480, LOVO, and HT29) following treatment with 5-aza-dC. Data are presented as mean ± SD. Statistical significance was determined by an unpaired two-tailed Student’s *t* test. ∗*p* < 0.05; ∗∗*p* < 0.01; ∗∗∗*p* < 0.001; and ∗∗∗∗*p* < 0.0001.

IHC was performed to assess SFMBT2 protein expression in FFPE tissue samples,^20^ including 111 primary CRC cases, 44 AA, 127 matched normal intestinal mucosae, 54 lymph node metastases, and 36 liver metastases. The findings indicated that SFMBT2 protein was robustly expressed in normal intestinal mucosa but was markedly diminished or absent in AA, primary CRC tissues, and metastatic lesions (Figures 1A and 1B). Quantitative analysis revealed that SFMBT2 expression in normal mucosa was significantly higher than in AA, primary CRC, and metastases (Table S1). Conversely, no significant differences in SFMBT2 expression were detected among AA, primary CRC, lymph node metastases, and liver metastases (*p* > 0.05). Additionally, SFMBT2 expression did not exhibit significant associations with patient age, sex, tumor differentiation, clinical stage, or tumor location (Table S2).

RT-qPCR analysis revealed that *SFMBT2* mRNA was highly expressed in low-metastatic CRC cell lines (HT29 and SW480) as well as in FHC cells, but was expressed at lower levels in highly metastatic cell lines (Lovo and SW620) (Figure 1C). Upon treatment with 5-aza-dC, *SFMBT2* mRNA expression was upregulated in all CRC cell lines, with the most pronounced increase observed in SW480 and HT29 cells (Figure 1D). These results suggest that *SFMBT2* expression is regulated by DNA methylation, and its downregulation in highly metastatic CRC cells may contribute to tumor progression, indicating a potential tumor-suppressive role for *SFMBT2* in CRC.

### The methylation levels of the *SFMBT2* gene in the colorectal cancer tissues

**Figure 2:**
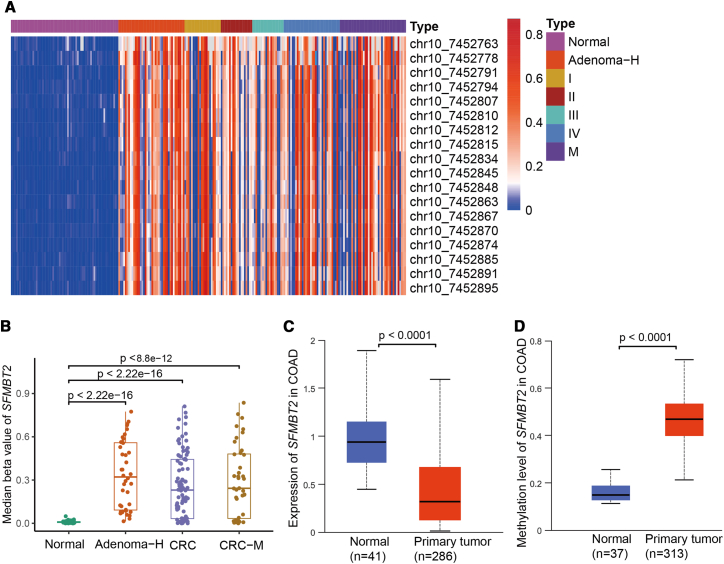
SFMBT2 promoter hypermethylation and gene expression levels in colorectal tissues (A and B) Targeted bisulfite sequencing reveals significant *SFMBT2* promoter hypermethylation in AA, primary CRC tissues, and distant metastatic lesions, compared with hypomethylated normal intestinal mucosa (*p* < 0.0001). (C) SFMBT2 mRNA expression was higher in normal mucosa than in CRC tissues (*p* < 0.0001) in TCGA. (D) SFMBT2 promoter methylation was higher in CRC tissues than in normal mucosa in TCGA (*p* < 0.0001). Data are presented as mean ± SD.

Targeted methylation sequencing was performed on 65 normal mucosa samples, 40 AA, 134 CRC samples, and 40 distant metastases (Table 1). The detection of the targeted methylation of *SFMBT2* encompasses 18 CG sites situated within the genomic region ranging from 7,452,763 to 7,452,895 on chromosome 10. The results revealed that the *SFMBT2* gene was hypomethylated in normal mucosa but significantly hypermethylated in AA, CRC tissues, and distant metastases (Figures 2A and 2B). Notably, no significant differences in methylation levels were observed among AA, CRC, and metastatic lesions, which aligns with the observed downregulation of SFMBT2 protein expression in these tissues. Also, no significant differences in *SFMBT2* levels were detected between 27 paired CRC tissues and their corresponding liver metastases (*p* > 0.05). Further analysis using data from TCGA confirmed that *SFMBT2* expression was significantly higher in normal intestinal mucosa compared to CRC tissues, while promoter methylation levels were markedly lower in normal tissues (Figures 2C and 2D). These findings demonstrate a strong inverse correlation between *SFMBT2* promoter methylation and gene expression, supporting the hypothesis that DNA hypermethylation may inhibit *SFMBT2* expression in CRC.

**Table 1:** Clinical characteristics of the tissue and plasma cohort

Sample	Tissue	Tissue	Tissue	Plasma	Plasma	
Characteristics	Normal	AA	CRC	Normal	AA	CRC
Total (n)	65	40	134	58	75	263
Sex						
Male	36(55%)	28(70%)	86((64%)	35(60%)	59(79%)	165(63%)
Female	29(45%)	12(30%)	48(36%)	23(40%)	16(21%)	98(37%)
Age (years)	57(22–68)	54(36–72)	62(47–79)	52(22–71)	56(32–80)	56(25–86)
≥50	43(66%)	29(72.5%)	107(80%)	34(59%)	54(72%)	208(79%)
<50	22(34%)	11(27.5%)	27(20%)	24(41%)	21(28%)	55(21%)
Stage						
Ⅰ	NA		22(15%)	NA		53(20%)
Ⅱ	NA		19(14%)	NA		66(25%)
Ⅲ	NA		19(14%)	NA		60(23%)
Ⅳ	NA		34(25%)^b^	NA		84(32%)
Hepatic metastasis^a^	NA		40(32%)	NA		
CEA quantification						
CEA≥ 5 ng/ml	NA	3(7.5%)	57(42%)	0	5(7%)	97(37%)
CEA< 5 ng/ml	NA	37(92.5%)	77(58%)	50	70(93%)	166(63%)

AA, Advanced adenoma.

a Hepatic metastatic tissues from patients with stage IV CRC.

b 27 paired Ⅳ stage CRC tissues and their hepatic metastasis.

### *SFMBT2* methylation levels detection in the plasma of patients with colorectal cancer

**Figure 3:**
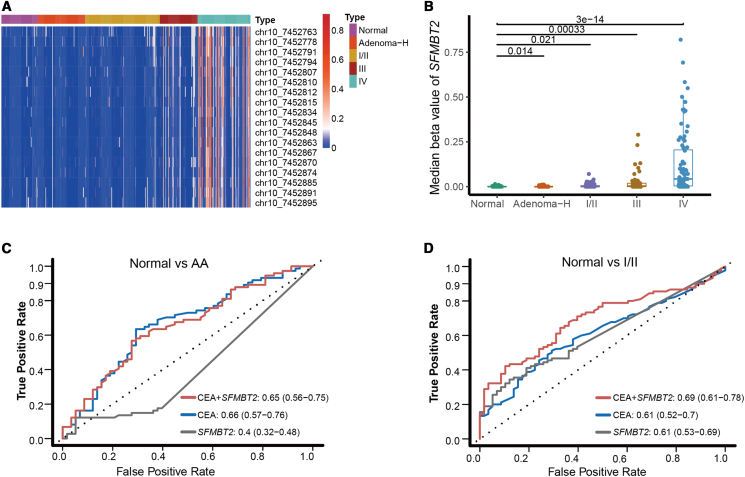
Plasma SFMBT2 methylation levels across different clinical stages of CRC (A) Heatmap shows elevated SFMBT2 methylation in ctDNA from stage IV and some patients with stage III CRC, with lower levels in normal controls, AA, and early-stage CRC (stage I–II). (B) Quantitative analysis confirmed significantly higher methylation levels in patients with advanced CRC (*p* < 0.001). (C) ROC curves for distinguishing AA from normal controls using SFMBT2 methylation, CEA, and their combination. (D) ROC curves for distinguishing early-stage CRC from normal controls using SFMBT2 methylation, CEA, and their combination. Data are presented as mean ± SD.

Targeted methylation sequencing of plasma samples revealed that *SFMBT2* methylation levels were very low in normal individuals. Patients with AA and early-stage (stage I/II) CRC exhibited significantly higher plasma methylation levels compared to normal controls (*p* < 0.05), although the overall methylation levels remained relatively low. However, patients with stage IV CRC and a subset of stage III patients exhibited markedly elevated *SFMBT2* methylation signals, which were significantly higher than those observed in normal controls, AA, and early-stage CRC (*p* < 0.001; Figures 3A and 3B). We constructed a random forest model to explore the efficacy of *SFMBT2* methylation in diagnosing AA and early-stage CRC. The area under the ROC curve (AUC) was 0.4 for AA and 0.61 for early-stage CRC, both of which are inferior to carcinoembryonic antigen (CEA), which had AUC values of 0.66 for AA and 0.61 for early-stage CRC. Specifically, *SFMBT2* methylation achieved a sensitivity of 12.2% and a specificity of 94.8% for AA detection, and a sensitivity of 34.4% and a specificity of 89.7% for early-stage CRC detection. When *SFMBT2* methylation was combined with CEA for distinguishing normal controls from AA and early-stage CRC, the AUC values remained at 0.65 for AA and 0.69 for early-stage CRC, respectively (Figures 3C and 3D). Given that several non-invasive methylation models have already been established for early CRC detection, which is significantly superior to *SFMBT2*.^21^^,^^22^ These results suggest that plasma *SFMBT2* methylation levels may not be ideal for CRC early screening. Because plasma *SFMBT2* methylation is increased in stage III CRC and is more significantly elevated in stage IV, we speculate that it may potentially reflect tumor burden in patients with CRC. Therefore, the potential of *SFMBT2* methylation in assessing recurrence risk and prognosis was further explored in patients with advanced CRC.

### Plasma *SFMBT2* methylation as a predictor of recurrence risk in patients with stage III colorectal cancer and prognosis in patients with stage IV colorectal cancer

**Figure 4:**
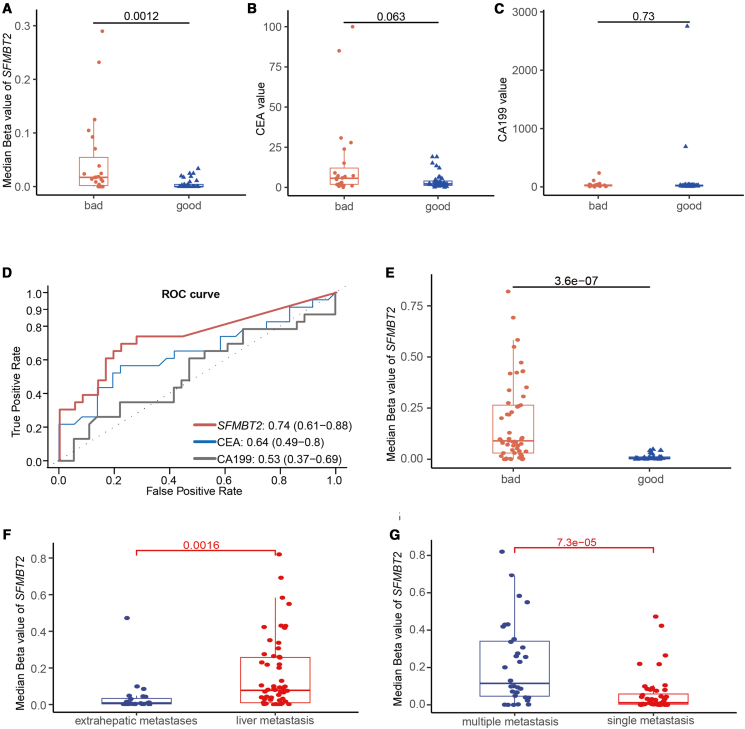
Prognostic value of plasma *SFMBT2* methylation in patients with stage III/IV CRC (A) Comparison of plasma SFMBT2 methylation levels between patients with recurrent and non-recurrent stage III CRC. (B and C) Comparison of plasma CEA (B) and CA19-9 (C) levels between patients with recurrent and non-recurrent stage III CRC. (D) ROC curves of SFMBT2 methylation, CEA, and CA19-9 for predicting recurrence in stage III CRC. (E) Plasma SFMBT2 methylation levels in patients with stage IV CRC with rapid vs. slow progression. (F) Comparison of SFMBT2 methylation levels between patients with liver and extrahepatic metastases. (G) SFMBT2 methylation levels in patients with single-site vs. multiple-site metastases. “good” = non-recurrent, “bad” = recurrent within 5 years. Data are presented as mean ± SD.

Plasma *SFMBT2* methylation levels were assessed in patients with stage III and IV CRC. Patients at stage III who experienced recurrence or disease progression within 5 years exhibited significantly higher methylation levels than those with stable disease (*p* = 0.001, Figure 4A). In contrast, no significant differences were observed in serum CEA and CA19-9 levels between the two groups (*p* > 0.05, Figures 4B and 4C). ROC curve analysis revealed that plasma *SFMBT2* methylation outperformed CEA and CA19-9 in predicting recurrence or progression in stage III CRC, with an AUC of 0.74 compared to 0.64 and 0.53 for CEA and CA19-9, respectively (Figure 4D). Among patients with stage IV CRC, those who developed recurrence or progression within 12 months after treatment (defined as rapid progressors) showed markedly elevated *SFMBT2* methylation compared to those with recurrence after ≥12 months (slow progressors) (*p* = 3.6 × 10^−7^, Figure 4E). Additionally, patients with liver metastases had significantly higher methylation levels than those with extrahepatic metastases (e.g., peritoneal, lung) (*p* = 0.0016, Figure 4F), and patients with multiple metastatic sites exhibited higher methylation than those with a single metastatic site (*p* = 7.3 × 10^−5^, Figure 4G). Collectively, these findings indicate that plasma *SFMBT2* methylation is a promising biomarker for predicting recurrence in patients with stage III CRC and may serve as a valuable prognostic indicator in patients with stage IV CRC.

### Plasma *SFMBT2* methylation dynamically reflects disease changes in patients with colorectal cancer

**Figure 5:**
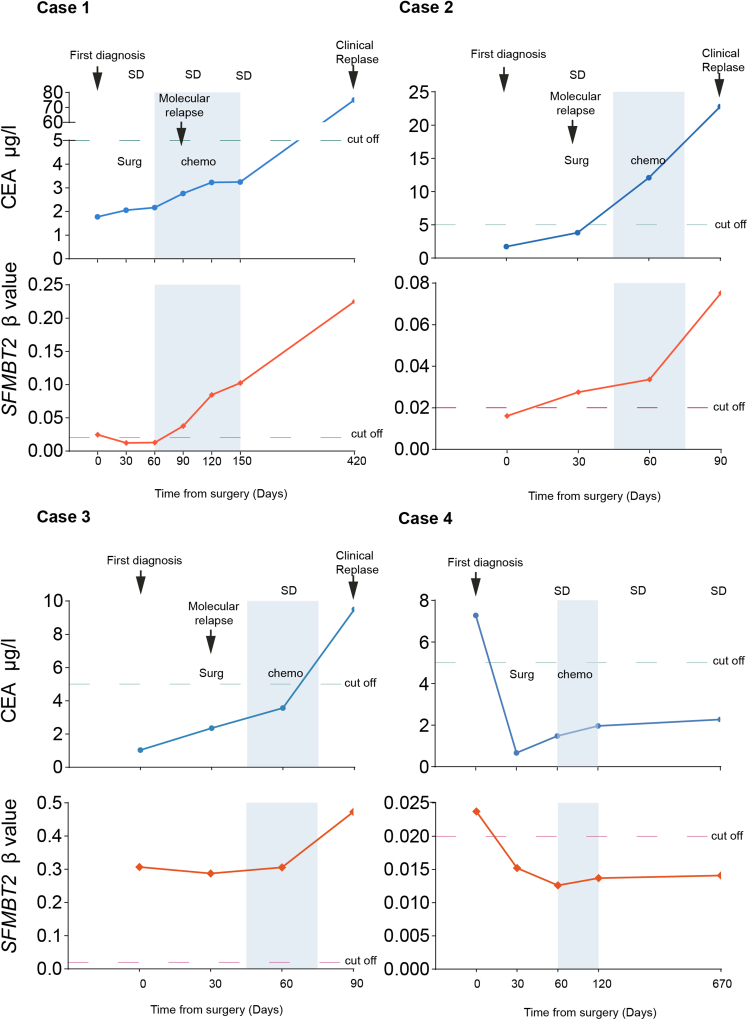
Dynamic monitoring of CRC progression and treatment response using plasma SFMBT2 methylation Case 1 shows a marked increase in SFMBT2 methylation approximately 12 months before serum CEA elevation and radiologic detection of recurrence. In Cases 2 and 3, methylation levels rose alongside tumor progression, serving as a useful supplement to CEA. Case 4 exhibited consistently low SFMBT2 methylation levels, corresponding with the stable disease.

Serial plasma samples from four patients with CRC were collected at three time points: preoperatively, postoperatively, and during follow-up treatment (Table S3), to investigate the potential of *SFMBT2* methylation as a dynamic biomarker for disease monitoring. The results demonstrated that plasma *SFMBT2* methylation levels reflected disease progression and treatment response. Notably, in Patient 1, a sharp increase in methylation levels occurred 12 months prior to serum CEA and radiological detection (Figure 5). In patients 2 and 3, methylation levels increased in parallel with tumor progression, while in patient 4, stable methylation levels corresponded with disease stability. However, the sample size is limited; these findings only reflect the preliminary observation. Whether *SFMBT2* methylation could serve as a complementary tool to serum CEA for monitoring disease status needs more consecutive samples to support.

### The role of *SFMBT2* in the immune microenvironment and metabolic regulation of colorectal cancer based on database analysis

**Figure 6:**
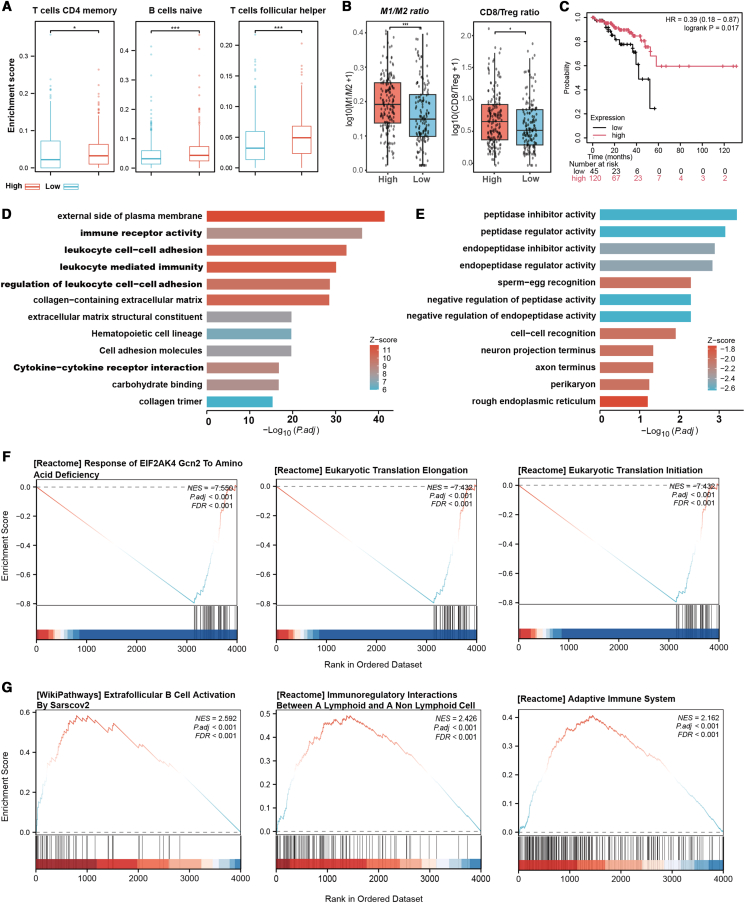
Tumor microenvironment characteristics and functional enrichment analysis associated with *SFMBT2* expression in CRC (A) Comparison of immune cell infiltration levels between SFMBT2 high- and low-expression groups, including CD4 memory activated T cells, naive B cells, and follicular helper T cells. (B) Comparison of M1/M2 ratio and CD8/Treg ratio between SFMBT2 high and low expression groups. (C) Kaplan-Meier survival analysis compares overall survival between SFMBT2 high and low-expression groups. (D) GO and KEGG pathway enrichment analysis of genes upregulated in the SFMBT2 high-expression group. (E) GO and KEGG pathway enrichment analysis of genes downregulated in the SFMBT2 high-expression group. (F) GSEA reveals significantly downregulated pathways in the SFMBT2 high-expression group. (G) GSEA reveals significantly upregulated pathways in the SFMBT2 high-expression group. Data are presented as mean ± SD. Statistical significance was determined by an unpaired two-tailed Student’s *t* test. ∗*p* < 0.05; ∗∗*p* < 0.01.

In patients with CRC with high *SFMBT2* expression, the infiltration levels of naive B cells, activated CD4^+^ memory T cells, and follicular helper T cells (Tfh) are significantly increased (Figure 6A). The M1/M2 ratio and CD8/Treg ratio in the *SFMBT2* high-expression group were both significantly higher than those in the low-expression group (both *p* < 0.05) (Figure 6B), suggesting that high *SFMBT2* expression may be associated with a stronger anti-tumor immune status. Further Kaplan-Meier survival analysis showed that patients with high *SFMBT2* expression had significantly better overall survival than those with low expression (*p* < 0.05) (Figure 6C), indicating that *SFMBT2* may influence prognosis by modulating the tumor immune microenvironment. Functional enrichment analysis shows that the upregulated genes in *SFMBT2* -high patients are mainly involved in immune receptor activity, leukocyte cell-cell adhesion, leukocyte-mediated immunity, and cytokine-cytokine receptor interaction (Figure 6D). In patients with colorectal cancer with high *SFMBT2* expression, the downregulated genes are mainly enriched in protease and protease regulator-related pathways (Figure 6E). This suggests that *SFMBT2* may shape the CRC immune microenvironment by influencing immune cell infiltration, regulating immune-related signaling pathways and protein degradation mechanisms, thereby participating in immune evasion or anti-tumor immunity. GSEA analysis showed that several immune activation pathways, such as adaptive immune response, immune cell interaction, and B cell activation, were enriched in *SFMBT2*-high patients (Figure 6F). Also, amino acid starvation response and eukaryotic translation-related pathways were significantly suppressed in the *SFMBT2*-high group (Figure 6G), hinting that *SFMBT2* might be involved in nutrient stress sensing and protein synthesis regulation.

## Discussion

Multiple studies have explored the utility of plasma-based single-gene or multi-gene methylation biomarkers for monitoring tumor burden and disease progression in patients with metastatic colorectal cancer (mCRC). For instance, Garrigou et al.^23^ demonstrated that the plasma methylation of *WIF1* and *NPY* detected 80% of mCRC cases and 45% of localized CRCs. Similarly, serum methylation of *HLTF* or *HPP1* has been identified as an independent prognostic marker in mCRC, with *HLTF* hypermethylation also serving as a predictor of CRC recurrence.^24^^,^^25^ The *SFMBT2* gene emerged as a promising biomarker candidate in two of our previous studies, which established plasma-based methylation models for CRC detection and recurrence evaluation.^9^^,^^10^ Jian-Bing Fan et al. also identified *SFMBT2* as part of a CRC-specific methylation panel for the early CRC detection.^18^ These findings collectively prompted us to further investigate the clinical utility of plasma *SFMBT2* methylation as well as the biological function of *SFMBT2* in colorectal tumorigenesis.

Bioinformatics analyses identified *SFMBT2* as a putative tumor suppressor gene. Studies showed that *SFMBT2* could inhibit tumor growth and metastasis in prostate cancer and clear cell renal cell carcinoma.^13^^,^^17^ Experimental validation revealed high *SFMBT2* mRNA expression in normal intestinal epithelial cells and low-metastatic CRC cell lines, whereas markedly reduced expression was observed in high-metastatic cell lines. This expression pattern is consistent with the inactivation of classical tumor suppressor genes such as *APC* and *TP53* in CRC.^26^^,^^27^ Treatment with the DNA demethylating agent 5-aza-dC significantly restored *SFMBT2* expression, particularly in HT29 and SW480 cells, indicating that promoter hypermethylation may be involved in the transcriptional regulation of *SFMBT2*. IHC further confirmed strong SFMBT2 protein expression in normal intestinal mucosa, with significant downregulation in AA, primary CRCs, and distant metastases. Notably, no significant differences in expression were observed among the latter three groups. Consistently, the tissue-level *SFMBT2* methylation assays showed no discernible differences among AA, primary CRC, and matched metastases. The TCGA analysis also demonstrated that *SFMBT2* expression is significantly higher in normal mucosa than in CRC tissues. In contrast, *SFMBT2* promoter methylation is low in normal tissues but markedly elevated in CRC tissues. This gene expression pattern and its inverse correlation with *SFMBT2* methylation status suggest that *SFMBT2* may act as a tumor suppressor gene, and methylation-mediated silencing is likely one of the main inactivation mechanisms of *SFMBT2* in CRC development. Additionally, it is widely recognized that the CpG island methylator phenotype (CIMP) plays a significant role in the development and progression of CRC.^28^^,^^29^ It is hypothesized that *SFMBT2* may contribute to the formation of CIMP by influencing the methylation status of CpG islands. However, further studies are required to discuss its specific mechanisms and clinical significance.

Research on *SFMBT2* has predominantly focused on its role in chondrocyte development and differentiation, with limited investigation into its role in cancer biology.^12^ A study identifying DMRs in human papillomavirus (HPV)-related oropharyngeal squamous cell carcinoma (OPSCC) revealed that *SFMBT2* hypermethylation can promote the migration and invasion of OPSCC cells by epigenetically silencing their transcriptional activity.^30^ In prostate cancer, *SFMBT2* suppresses metastasis by modulating matrix metalloproteinases *MMP-9* and *MMP-26* and has been proposed as a potential biomarker for metastatic disease.^13^ These findings collectively suggest that *SFMBT2* may serve as a diagnostic or prognostic biomarker in various cancer types, warranting further investigation in CRC.

Therefore, we further investigated the clinical diagnostic and prognostic significance of *SFMBT2* methylation using plasma sequencing data from patients with CRC. When applied to plasma *SFMBT2* for the diagnosis of AA and early-stage (stages I-II) CRC, the AUC was only 0.40 and 0.61, respectively. These values were inferior to those of serum carcinoembryonic antigen (CEA), which had AUC values of 0.66 and 0.61. Even when combining plasma CEA and SFMBT2 methylation, the diagnostic performance did not significantly improve. As is well known, the Epi proColon assay *SEPT9* yielded a sensitivity of 73% at a specificity of 94.5% for CRC detection in a prospective clinical trial.^31^ A single methylation biomarker, *cg10673833*, was demonstrated to be a superior methylation biomarker in CRC detection, with a sensitivity and specificity of 89.7% and 86.8%, respectively.^32^ Therefore, the *SFMBT2* methylation plasma alone is insufficient for the early screening of CRC; it is frequently incorporated into combined methylation panels for enhanced detection.

However, *SFMBT2* methylation levels are significantly elevated in advanced-stage CRC (stage III/IV). Among patients with stage III disease, increased plasma *SFMBT2* methylation was associated with a higher risk of relapse and worse prognosis (AUC = 0.74), which outperforms traditional serum markers CEA and CA199, indicating its potential to identify patients with high-risk stage Ⅲ CRC that need intensified therapy. Similarly, in patients with stage IV CRC, those with rapidly progressing disease exhibited significantly higher plasma *SFMBT2* methylation levels compared to those with slower disease progression. Moreover, *SFMBT2* hypermethylation was significantly associated with metastatic patterns. Specifically, patients with liver metastases had higher methylation levels than those with extrahepatic metastases. Additionally, the number of metastases correlated with *SFMBT2* methylation levels, with higher levels observed in patients with multiple metastases compared to those with single lesions. *SFMBT2* methylation may serve as a potential indicator for monitoring the recurrence risk and prognosis of patients with advanced CRC.

Longitudinal plasma analyses from four patients further demonstrated that *SFMBT2* methylation outperformed conventional serum markers, such as CEA, in monitoring disease progression. It not only dynamically reflected treatment responses but also predicted recurrence up to 12 months earlier than the CEA test. Despite the relatively small sample size, our findings suggest that *SFMBT2* methylation may serve as a promising biomarker for dynamic disease monitoring. Similarly, dynamic monitoring of *NPY* methylation levels can assess therapeutic efficacy in metastatic colorectal cancer (mCRC) and predict disease progression. Notably, a significant increase in *NPY* methylation levels was detected 49 days earlier than radiological progression.^33^^,^^34^ Further verification is needed to determine whether these biomarkers can serve as a promising complementary tool to the current surveillance system.

In colorectal cancer, high *SFMBT2* expression is associated with the increased infiltration of various immune cells, including activated CD4^+^ memory T cells, naive B cells, Tfh cells, M1 and M2 macrophages, CD8^+^ T cells, and Tregs. Despite the presence of both pro-tumor and anti-tumor cell populations, ratio analyses (e.g., M1/M2 and CD8/Treg ratios) and Kaplan-Meier survival analysis indicate that high *SFMBT2* expression is linked to a stronger anti-tumor immune response and better overall survival. Functional enrichment and GSEA analyses supported these observations, showing the enrichment of immune signaling pathways and enhanced cell-cell interaction processes in the high-expression group. Taken together, these results suggest that *SFMBT2* expression may be associated with the modulation of immune activity in CRC. However, it is important to note that these conclusions are correlative and exploratory, as they are based on computational analyses rather than direct experimental validation. Further studies, including immunohistochemistry and multiplex immunofluorescence, will be required to confirm these associations and clarify their mechanistic basis.

In summary, *SFMBT2* may act as a tumor-suppressor gene in CRC development, and methylation may be implicated in its transcriptional regulation. Plasma *SFMBT2* methylation detection demonstrates significant potential as a liquid biopsy marker for recurrence monitoring and prognostic evaluation of advanced CRC. The potential of *SFMBT2* methylation as a biomarker for dynamic disease monitoring needs more validation. Its superior performance in risk stratification effectively compensates for the limitations of current biomarkers, positioning it as a valuable supplementary tool in clinical practice. Additionally, *SFMBT2* may influence CRC biology through immune microenvironment regulation. This study provides molecular insights to guide precision diagnosis, stratified therapeutic interventions, and targeted immunotherapy development for patients with advanced CRC.

### Limitations of the study

The biological functions of *SFMBT2* in CRC require further mechanistic studies. The efficacy of plasma *SFMBT2* for the dynamic monitoring of CRC needs to be verified with more consecutive samples. Although current studies indicate that *SFMBT2* is not suitable for the early screening of CRC, its combined application value with other markers (such as ctDNA and CEA) needs to be explored.

## Resource availability

### Lead contact

Further information and requests for resources and reagents should be directed to the lead contact: Please contact Wei Wang at ricewang79@126.com for data requests.

### Materials availability

This study did not generate new unique reagents.

### Data and code availability

#### Data

Publicly available transcriptomic data of patients with colorectal cancer analyzed in this study were obtained from The Cancer Genome Atlas (TCGA) database, including the TCGA-COAD and TCGA-READ cohorts (see key resources table).

The data reported in this article have been deposited in the OMIX database, China National Center for Bioinformation/Beijing Institute of Genomics, Chinese Academy of Sciences (https://ngdc.cncb.ac.cn/omix; accession No. OMIX014633) (see key resources table). In accordance with national regulations on the sharing of human genetic resources, the data are available under controlled access. Researchers may apply for access through the OMIX data access request system following the official OMIX data access guidelines, and approved data may be used for research purposes only.

#### Code

This study did not generate new custom code. All analyses were performed using publicly available software and R packages, which are listed in the key resources table.

#### Other items

Any additional information required to reanalyze the data reported in this article is available from the lead contact upon reasonable request.

## Acknowledgments

The authors thank all patients who participated in this study.

This work was supported by the 10.13039/501100003453Natural Science Foundation of Guangdong Province of China (NO. 2023A1515012384) and the Science and Technology Planning Project of Guangzhou (NO. 2023A03J0169).

## Author contributions

W.W. and L.Y. carried out experiments. J.W. and Y.L. performed the statistical analysis and drew the schematic diagrams. W.C., W.H., and J.Z. assisted in collecting tissue and plasma samples and following up on the cases. X.L. and D.Y. helped carry out some experiments. C.L. conceived the experiments and wrote the article. All authors were involved in writing the article and had final approval of the submitted and published versions.

## Declaration of interests

The authors declare no competing interests.

## STAR★Methods

### Key resources table

**Table:** 

REAGENT or RESOURCE	SOURCE	IDENTIFIER
**Antibodies**		

Rabbit anti-human SFMBT2 polyclonal antibody	Proteintech	25256-1-AP; RRID: AB_2833000
Anti-rabbit IgG secondary antibody	Dako	Cat# P0217

**Biological samples**		

Human colorectal cancer tissue samples	This study	–
Human plasma samples	This study	–

**Chemicals, peptides, and recombinant proteins**		

5-aza-2′-deoxycytidine (5-aza-dC)	Sigma-Aldrich	A3656

**Critical commercial assays**		

Trizol reagent	Invitrogen	15596026
Revertaid First Strand cDNA Synthesis Kit	Thermo Scientific	K1621
SYBR Green qRT-PCR Master Mix	TaKaRa	RR430S / RR430A
EZ DNA Methylation-Lightning Kit	Zymo Research	Cat# D5031
AnchorDx EpiVisio™ Methylation Library Prep Kit	AnchorDx	Cat# A0UX00019
AnchorDx EpiVisio™ Indexing PCR Kit	AnchorDx	Cat# A2DX00025
AnchorDx EpiVisio™ Target Enrichment Kit	AnchorDx	Cat# A0UX00031
Acellular DNA BCT® tubes	Streck	Cat# 218962
QIAamp DNA FFPE Tissue Kit	Qiagen	Cat# 56404
Bioo NextPrep-Mag™ cfDNA Isolation Kit	Bioo Scientific	Cat# NOVA-3825
Agilent High Sensitivity DNA Kit	Agilent Technologies	Cat# 5067-4626
Qubit™ dsDNA HS Assay Kit	Thermo Fisher Scientific	Cat# Q32854

**Deposited data**		

TCGA-COAD and TCGA-READ transcriptomic data	The Cancer Genome Atlas	https://portal.gdc.cancer.gov/
TCGA-COAD and TCGA-READ DNA methylation array data	The Cancer Genome Atlas	https://portal.gdc.cancer.gov/
DNA methylation beta value matrices	This study	No. OMIX014633

**Experimental models: Cell lines**		

HT29	ATCC	Cat# HTB-38
SW480	ATCC	Cat# CCL-228
SW620	ATCC	Cat# CCL-227
LoVo	ATCC	Cat# CCL-229
FHC	ATCC	Cat# CRL-1831

**Software and algorithms**		

R	R Foundation	v3.6.0
SPSS Statistics	IBM	v25.0
GraphPad Prism	GraphPad Software	v9.5
limma	Bioconductor	–
pROC	CRAN	v1.15.3
ggplot2	CRAN	V3.4.2
ComplexHeatmap	Bioconductor	v2.14.0
CIBERSORT	Newman et al.	https://cibersort.stanford.edu
GSEA	Broad Institute	–
MSigDB	Broad Institute	https://www.gsea-msigdb.org

### Experimental model and study participant details

#### Human subjects

Tissue and plasma samples were obtained from CRC patients at the General Hospital of the Southern Theater Command and the Southern Hospital of Southern Medical University. The inclusion criteria were as follows: (1) pathologically confirmed primary CRC diagnosis; (2) stage IV CRC patients who had undergone curative-intent metastasectomy for liver, lung, or peritoneal metastases; (3) availability of analyzable tissue and/or plasma samples; and (4) a tumor cell proportion of no less than 30% in FFPE samples. The exclusion criteria were: (1) a history of preoperative treatments; (2) incomplete clinical data; and (3) a personal history of other malignancies. Advanced adenomas were defined as adenomas with a size≥1 cm, high-grade dysplasia, or villous or tubule-villous histology. Adjacent normal mucosal samples were collected from sites located more than 5 cm away from the primary tumor margin.

This study employed a dual-cohort approach using FFPE tissue samples to systematically investigate *SFMBT2* expression and methylation patterns across different stages of CRC, as well as in normal and metastatic tissues. For immunohistochemical detection, a total of 372 FFPE tissue samples were collected, including 44 AA, 111 primary tumors, 127 matched normal mucosa samples, 54 lymph node metastases, and 36 liver metastases. Furthermore, targeted methylation sequencing was conducted on a cohort of 239 FFPE tissue samples which included 65 normal mucosae, 40 AA cases, 22 stage I CRC samples, 19 stage II and 19 stage III CRC samples, 34 stage IV CRC, and 40 distant metastatic lesions (27 paired stageⅣ CRC tissues and its hepatic metastasis). This cohort included 75 patients with AA, and 263 CRC patients across different TNM stages. Healthy controls (n=58) were defined as individuals over 18 years without prior cancer history or chronic diseases. The FFPE slides and plasma samples derived from different patients. The detailed clinical data for issue and plasma samples are summarized in Table 1.

#### Ethics

The participants were enrolled in Southern Hospital and General Hospital of Southern Theater Command and Southern Hospital of Southern Medical University, and written informed consent was obtained from all patients before tissue and blood samples collection. All participants were informed of the usage of tissues and plasma and the test results. The current study was approved by Ethics committee of Southern Hospital (No. 2020-010) and General Hospital of Southern Theater Command (No.2024GJJ036).

### Method details

#### Cell lines culture

Human CRC cells (HT29, SW480, SW620, and Lovo) and fetal human colon (FHC) cells were all procured from the American Type Culture Collection (ATCC). HT29 belongs to the CIMP-positive^35^ cell lines. 2.5×10^6^ cells were seeded in 6-well plates in RPMI-1640 (Gibco) medium supplemented with 10% FBS and 1% penicillin single bondstreptomycin. The cells were cultured at 37 °C in a humidified atmosphere containing 95% air and 5% CO2. All cell lines were cultured for no more than 20 passages, verified negative for mycoplasma contamination using the MycoAlert Mycoplasma Detection Kit (Lonza Bioscience), and authenticated by short tandem repeat (STR) genotyping analysis. STR profiles were generated for all cell lines and aligned with the standard databases of ATCC and DSMZ, with a matching degree of ≥95% confirmed for each cell line.

#### RNA extraction

For demethylation treatment, cells were treated with 10 μM 5-aza-2’-deoxycytidine^36^ (5-aza-dC) for 72 h, followed by RNA extraction. Total RNA was isolated from the human CRC cells using the Trizol-chloroform extraction method as described by the manufacturers. The total cDNA was reverse-transcribed using the Revertaid First Strand cDNA Synthesis Kit (Thermo Scientific, USA).

#### Quantitative real-time PCR

The specific primers for *SFMBT2* that were used in RT-qPCR were designed using Primer Premier 5.0 and produced by Sangon Biotech Co., Ltd. (Shanghai, China). GAPDH was used as the housekeeping gene. The primer sequences were as follows: *SFMBT2* forward, 5'-AAAAGTGTCTCGGCTCAGCTA-3' and reverse, 5'-ACGTGTCCGG GTTGTTCTTAT-3' GAPDH forward, 5'-ATGCTGGAAAACCTTGAGCTT-3' and reverse, 5'-TGGAAGGATCTGCATTAGGGA 3’. qRT-PCR was performed in a 7500 Fast Real-Time PCR System with SYBR GreenqRT-PCR master mix (TaKaRa).

#### Immunohistochemistry

The rabbit anti-human polyclonal antibody SFMBT2 (final dilution 1:400) was purchased from Proteintech. The FFPE specimens were cut into 4-μm-thick sections. The SFMBT2 immunohistochemical staining was performed on Dako automatic immunohistochemistry instrument following the manufacturer' instructions, briefly including blocking endogenous peroxidase, adding primary antibody, washing the slides, adding anti-rabbit IgG (Dako), chromogenic detection, counterstaining, dehydration, and clearing. Negative control sections were incubated with PBS instead of primary antibody. The sections were independently examined by two pathologists over 15 years of experience, who were blinded to the clinicopathological information. SFMBT2 immunohistochemical staining was localized in the cytoplasm and membrane of cells, and the quantification criteria for the staining was performed as follows: with: Cells with <10% staining were scored as negative staining (−, 1); cells with 10-49% staining were scored as (+, 2); cells with 50-74% staining were scored as (++, 3); and cells with 75-100% staining were scored as (+++, 4). The staining color was scored as light-yellow particle (1), brown-yellow particle (2), and brown particle (3). The final score was defined as staining number score multiplied by staining color score.^20^ Samples with scores ≤5 were classified as negative, whereas those with scores >5 were classified as positive.

#### Plasma processing

Blood samples (10 mL) were collected using acellular DNA BCT® blood collection tubes (Streck, catalog 218962), and separated by centrifugation at 1600 rpm for 10 min at 4 °C (Beckman Xllegra X-12R), followed by a second centrifugation at 16000 rpm for 10 min at 4 °C (GeneSpeed GS-1730R), and stored at -80 °C until DNA separation.

#### High-throughput targeted methylation sequencing

Bisulfite conversion^37^ of genomic DNA was carried out using the EZ DNA Methylation-Lightning Kit (Zymo Research, Cat# D5031) according to the manufacturer’s instructions. Targeted methylation profiling was conducted using the proprietary AnchorIRIS™ technology (AnchorDx).^22^ For plasma samples, 10 ng of input cell-free DNA (cfDNA) was used for library construction. Libraries were prepared using the AnchorDx EpiVisio™ Methylation Library Prep Kit (AnchorDx, Cat# A0UX00019) and the AnchorDx EpiVisio™ Indexing PCR Kit (AnchorDx, Cat# A2DX00025). Prehybridization libraries with a total DNA amount exceeding 400 ng were considered qualified for target enrichment. Target enrichment was performed using the AnchorDx EpiVisio™ Target Enrichment Kit (AnchorDx, Cat# A0UX00031) together with a custom-designed cancer methylation panel. For each enrichment reaction, up to four prehybridization libraries (totaling 1,000 ng DNA) were pooled and captured using the PanMet V2 methylation panel, which targets 12,624 preselected CpG regions enriched for cancer-specific methylation events. The enriched libraries were sequenced on an Illumina HiSeq X Ten platform (Illumina, San Diego, CA, USA) using paired-end sequencing. Both tissue-derived DNA and plasma-derived cfDNA libraries were sequenced to an average on-target depth of approximately 5,000×, with approximately 20 million paired-end reads generated per sample.

#### Bioinformatics and transcriptomic analysis

Transcriptomic data of colorectal cancer (CRC) patients were obtained from The Cancer Genome Atlas (TCGA) database, including RNA-sequencing data from the TCGA-COAD and TCGA-READ cohorts. Gene expression data were downloaded in TPM format and log2-transformed prior to analysis. Patients were stratified into high- and low-expression groups based on the median expression level of *SFMBT2*. Immune cell infiltration in tumor tissues was estimated using the CIBERSORT^38^ algorithm with the LM22 gene signature matrix and 1,000 permutations. Only samples with CIBERSORT output *p* < 0.05 were included for downstream analyses. Differentially expressed genes (DEGs) between SFMBT2 high- and low-expression groups were identified using the “limma” R package,^39^ with thresholds of |log2 fold change| > 0.5 and false discovery rate (FDR) < 0.05. Gene Ontology (GO) and Kyoto Encyclopedia of Genes and Genomes (KEGG) pathway enrichment analyses were performed on significantly altered DEGs. Gene Set Enrichment Analysis (GSEA) was conducted using the GSEA software^40^ (Broad Institute) to assess differences in immune- and metabolism-related pathways between groups. Hallmark and KEGG gene sets were obtained from the Molecular Signatures Database (MSigDB),^41^ and gene sets with FDR < 0.25 were considered significantly enriched.

### Quantification and statistical analysis

All statistical analyses (including Chi-square test and Wilcoxon) were performed using R (version 3.6.0),SPSS 25.0 (IBM SPSS Statistics 25, IBM, Somers, IL, USA) and Graphpad Prism (Graphpad Prism 9.5). Significance was determined with a threshold of p<0.05. The receiver operating characteristic (ROC) curves were generated with the pROC R package^42^ (version 1.15.3) to evaluate the performance of *SFMBT2* methylation and CEA quantification in early diagnosis and predicting prognosis. The Chi-square test was used to compare the relationship between *SFMBT2* expression and clinicopathologic features in CRC. The Wilcoxon rank sum test was used to compare the methylation level and mRNA expression of SFMBT2 between the different groups. The sensitivity, specificity, and the corresponding 95% CI were also calculated using the “pROC” package. Bar plots, scatterplot, and other visualizations were generated using the “ggplot2” package (version 3.4.2). Heatmaps were created using the “Complex Heatmap” package (version 2.14.0). Data are presented as mean ± SD. Statistical significance was determined by unpaired two-tailed Student’s t test. ∗*p* < 0.05; ∗∗*p* < 0.01; ∗∗∗*p* < 0.001; ∗∗∗∗*p* < 0.0001.
