# Supplementary material for: SFMBT2 hypermethylation promotes colorectal cancer progression and is a potential noninvasive biomarker for advanced CRC
Source: iScience. 2026 Feb 13;29(3):115019. doi: 10.1016/j.isci.2026.115019 (PMC12969127; doi:10.1016/j.isci.2026.115019)
Supplement: Document S1. Tables S1–S3 [file mmc1.pdf]

iScience, Volume *29*

Supplemental information

SFMBT2 hypermethylation promotes colorectal cancer progression and is a potential noninvasive biomarker for advanced CRC

Wei Wang, Ling Yu, Wenyuan He, Jingyi Wu, Yuansen Li, Wenzhi Cui, Danli Ye, Juan Zhou, Ming Xie, Xuwen Lai, and Chengyong Lei

Table S1 Expressions of SFMBT2 in normal mucosa, adenoma, CRC tissues, lymphatic metastatic tissues and liver metastasis by IHC, Related to Figure 1.

Group	SFMBT2				Total	*P*
	-	+	++	+++		
Normal Mucosa	4	7	53	63	127	
Advanced Adenoma	5	14	23	4	46	<0.0001
Normal Mucosa	4	7	53	63	127	
Colorectal tissue	27	39	37	7	111	<0.0001
Stage I/II	18	19	16	0	53	<0.0001
Stage III/IV	9	21	21	7	58	<0.001
Normal Mucosa	4	7	53	63	127	
lymphaticMetastasis	9	19	19	7	54	<0.0001
Normal Mucosa	4	7	53	63	127	
Liver Metastasis	5	15	11	4	35	<0.0001

Table S2 Relationship between *SFMBT2* expression and clinicopathologic features in CRC, Related to Figure 1.

Features	Total	Low	High	*P*	λ^2
Age				0.358	0.366
<50	24	13	11		
≥50	57	35	22		
Gender				0.071	2.918
Male	53	35	18		
Female	28	13	15		
Differentiation				0.237	5.830
Well	24	14	10		
Moderate	44	30	14		
Poor	13	4	9		
Clinical Stages				0.082	6.887
I/II	39	25	14		
III	15	12	3		
IV	27	11	16		
Location				0.445	0.155
left colon	61	37	24		
Right colon	20	11	9		

Table S3 Clinical data of 4 cases of serial blood samples, Related to Figure 5.

Patient no.	Date	Disease course	TNM	CEA	CT	SFMBT2 value
**Case 1**						
CR006-001	170508	Pre-surgery	IIIb	1.77	Positive	0.0246
CR006-227	170515	Post-surgery	IIIb	2.05	Negative	0.0124
CR006-228	170618	Chemotherapy	IIIb	2.16	Negative	0.0129
CR006-229	170808	Chemotherapy	IIIb	2.76	Negative	0.0377
CR006-230	170903	Chemotherapy	IIIb	3.23	Negative	0.0845
CR006-231	171026	Chemotherapy	IIIb	3.24	Negative	0.1026
CR006-232	180725	Progressive disease	IVa	75	Positive	0.2250
**Case 2**						
CR006-025	170710	Pre-surgery	IVa	1.74	Positive	0.0161
CR006-235	170922	Post-surgery	IVa	3.83	Negative	0.0276
CR006-236	171013	Chemotherapy	IVa	12.1	Negative	0.0337
CR006-237	171216	Progressive disease	IVa	22.8	Positive	0.0753
**Case 3**						
CR006-057	170710	Pre-surgery	IIIb	1.03	Positive	0.3069
CR006-233	170814	Post-surgery	IIIb	2.36	Negative	0.2876
CR006-234	170926	Chemotherapy	IIIb	3.57	Negative	0.3055
CR006-239	171130	Progressive disease	IVa	9.5	Positive	0.4731
**Case 4**						
CR006-002	170509	Pre-surgery	IIIb	7.28	Positive	0.0237
CR006-421	170618	Post-surgery	IIIb	0.66	Negative	0.0152
CR006-422	170831	Chemotherapy	IIIb	1.48	Negative	0.0126
CR006-423	171123	Chemotherapy	IIIb	1.96	Negative	0.0137
CR006-424	190320	Stable disease	IIIb	2.27	Negative	0.0141
